# Single-cell transcriptome analysis of fish immune cells provides insight into the evolution of vertebrate immune cell types

**DOI:** 10.1101/gr.207704.116

**Published:** 2017-03

**Authors:** Santiago J. Carmona, Sarah A. Teichmann, Lauren Ferreira, Iain C. Macaulay, Michael J.T. Stubbington, Ana Cvejic, David Gfeller

**Affiliations:** 1 Ludwig Center for Cancer Research, University of Lausanne, 1066 Epalinges, Switzerland;; 2 Swiss Institute of Bioinformatics (SIB), 1015 Lausanne, Switzerland;; 3 European Molecular Biology Laboratory, European Bioinformatics Institute, Wellcome Trust Genome Campus, Hinxton, Cambridge CB10 1SD, United Kingdom;; 4 Wellcome Trust Sanger Institute, Wellcome Trust Genome Campus, Cambridge CB10 1SA, United Kingdom;; 5 Department of Haematology, University of Cambridge, Cambridge CB2 0XY, United Kingdom;; 6 Wellcome Trust–Medical Research Council, Cambridge Stem Cell Institute, Cambridge CB2 1QR, United Kingdom;; 7 Sanger Institute–EBI Single-Cell Genomics Centre, Wellcome Trust Genome Campus, Hinxton, Cambridge CB10 1HH, United Kingdom

## Abstract

The immune system of vertebrate species consists of many different cell types that have distinct functional roles and are subject to different evolutionary pressures. Here, we first analyzed conservation of genes specific for all major immune cell types in human and mouse. Our results revealed higher gene turnover and faster evolution of *trans*-membrane proteins in NK cells compared with other immune cell types, and especially T cells, but similar conservation of nuclear and cytoplasmic protein coding genes. To validate these findings in a distant vertebrate species, we used single-cell RNA sequencing of *lck:GFP* cells in zebrafish and obtained the first transcriptome of specific immune cell types in a nonmammalian species. Unsupervised clustering and single-cell *TCR* locus reconstruction identified three cell populations, T cells, a novel type of NK-like cells, and a smaller population of myeloid-like cells. Differential expression analysis uncovered new immune-cell–specific genes, including novel immunoglobulin-like receptors, and neofunctionalization of recently duplicated paralogs. Evolutionary analyses confirmed the higher gene turnover of *trans*-membrane proteins in NK cells compared with T cells in fish species, suggesting that this is a general property of immune cell types across all vertebrates.

The immune system of vertebrate species has evolved into a highly complex structure, comprising many different types of both innate and adaptive immune cells. Adaptive immune cells are broadly classified into B and T lymphocytes that can directly recognize antigens with great specificity. Innate immune cells include a variety of myeloid cells such as monocytes, neutrophils, basophils, eosinophils, and mast cells. A third major type of lymphocytes, the Natural Killer (NK) cells, has also been historically classified among innate immune cells (Sun and Lanier 2009; Sun et al. 2009). Traditionally, different immune cell types are distinguished based on unique combinations of cell surface markers. In mouse and human, many antibodies for these markers are available and can be used to isolate homogeneous immune cell populations using flow cytometry. Gene expression profiling studies of isolated immune cell populations have further allowed genome-wide identification of cell-type–specific genes (Chambers et al. 2007; Watkins et al. 2009; Shay and Kang 2013; Vu Manh et al. 2014). These studies revealed an overall conservation of immune cells’ gene expression between mouse and human (Shay et al. 2013). However, beyond mouse and human, less is known about the characteristics and evolution of immune cell types mainly due to the challenges of isolating different immune cell populations.

Evolutionary studies based on mouse and human genes have shown that immune-related genes tend to evolve faster than other genes (Kosiol et al. 2008; Flajnik and Kasahara 2010; Boehm 2012; Bailey et al. 2013). This faster evolution may reflect a need of immune cells to adapt to a rapidly changing environment and specific pathogens. In addition, different immune cell types are subject to different evolutionary constraints. T and B lymphocytes can generate an extraordinary diverse repertoire of antigen-specific receptors as a consequence of *Rag-*mediated somatic V(D)J (variable diversity joining) rearrangement, and this process is conserved across all jawed vertebrates (Boehm 2012). Many orthologs of T-cell–specific genes, like *CD4*, *CD8*, and *TCR* genes, have been identified in all jawed vertebrates. In species like zebrafish, the V(D)J variable regions have been recently annotated (Schorpp et al. 2006; Meeker et al. 2010; Iwanami 2014). NK receptors instead are germline-encoded. Therefore, selection pressure to generate different receptor specificities and transduce signals is expected to operate at the population rather than at the individual cell level. Indeed, mammalian NK-cell receptors have expanded and diversified in a species-specific fashion, as in the case of KIR receptors in primates and Ly49/killer cell lectin-like receptors in rodents (Carrillo-Bustamante et al. 2016). NK-like cells have been identified in nonmammalian species such as chicken (Jansen et al. 2010), *Xenopus* (Horton et al. 1996), and catfish, where spontaneous killing of allogeneic cells by non-TCR expressing cytotoxic cells was demonstrated (Shen et al. 2004; Yoder 2004). A recent study using single-cell qPCR based on known markers of blood cell lineages revealed the presence of a small population of immune cells in zebrafish, which were proposed to represent putative NK-like cells based on expression of NK-lysin genes (Moore et al. 2016). The identification of membrane receptors with similar genomic organization as the *KIR* genes in humans provided additional evidence for the existence of NK cells in fish species. In zebrafish, these receptors include *nitr* (Yoder et al. 2004) and *dicp* genes (Haire et al. 2012). However, pure T- and NK-cell populations have so far not been isolated in zebrafish, and no reliable antibody has been developed against orthologs of mammalian T- and NK-cell receptors. Therefore, many properties of mammalian T- and NK-cell orthologs and their evolution in nonmammalian species remain uncharacterized.

High-throughput single-cell RNA-seq (scRNA-seq) has emerged as a promising technology to unravel the landscape of cell types in heterogeneous cell populations without relying on specific antibodies (Saliba et al. 2014). The simultaneous expression of thousands of genes can be measured in each cell, thereby providing an unbiased view of transcriptional activity at the cellular level and avoiding the averaging effect of bulk gene expression studies (Shapiro et al. 2013). Cells can then be grouped into biologically relevant clusters based on the similarity of their gene expression profiles rather than a handful of cell surface markers (Grün et al. 2015; Trapnell 2015; Macaulay et al. 2016). Therefore, despite technical and biological noise and the computational challenges associated with this variability (Brennecke et al. 2013; Buettner et al. 2015), scRNA-seq has the potential to uncover new immune cell types that cannot be studied using traditional approaches.

To gain insight into the evolution of vertebrate immunity and nonmammalian immune cell types, we first analyzed the conservation of mouse and human immune cell (i.e., T-, B-, NK-, and myeloid cells)–specific genes. Next, we analyzed immune cells in zebrafish, a powerful model in biomedical research (Langenau and Zon 2005; Renshaw and Trede 2012; Kaufman et al. 2016). To this end, we took advantage of a transgenic line of zebrafish expressing GFP under the control of the *lck* promoter (Langenau et al. 2004) and performed scRNA-seq on *lck:GFP*^+^ FACS sorted cells.

## Results

### Conservation analysis of mammalian T-, B-, NK-, and myeloid-cell–specific genes across vertebrates

Immune related genes tend to evolve more rapidly than other genes, and between functionally distinct immune cells, the selective pressures might vary significantly. Here we performed a conservation analysis of the most differentially expressed genes in resting T, B, NK, and myeloid cells in the mouse and human at the genome-wide level (see Methods) (Chambers et al. 2007; Watkins et al. 2009).

**Figure 1.**
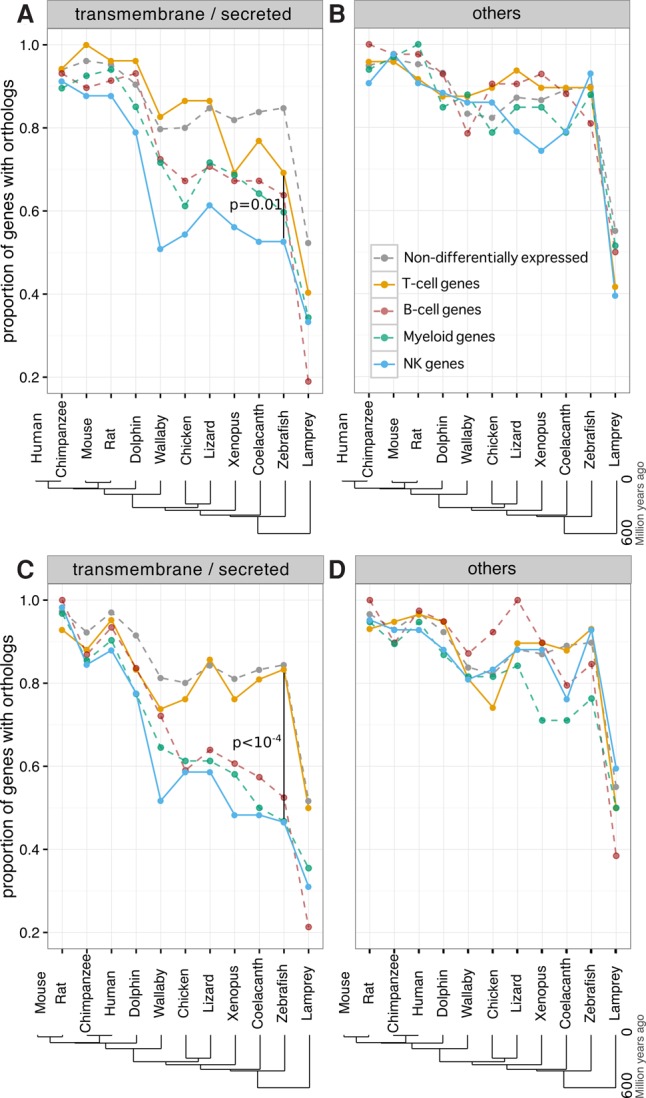
Conservation analysis of human and mouse genes differentially expressed in major immune cell types. (*A*,*B*) Proportion of human genes specific for distinct immune cell types (T, B, NK, and myeloid cells) with orthologs in other species. Results for genes coding for *trans*-membrane (TM) and secreted proteins (*A*) and for cytoplasmic and nuclear proteins (*B*). (*C*,*D*) Same analysis as in *A* and *B* using mouse immune cell types’ specific genes.

Our analysis revealed that among *trans*-membrane (TM) or secreted protein coding genes, those specifically expressed in NK cells have proportionally fewer orthologs across all vertebrates compared with other immune cells. The difference is most evident between NK and T cells, although these are closer from a functional and ontogenical point of view (Fig. 1A,C). No difference, however, was observed for cytoplasmic or nuclear protein coding genes (Fig. 1B,D). As expected, the killer cell lectin-like receptors in mouse and KIRs in human strongly contributed to this difference. Interestingly, however, the differences between T- and NK-cell TM gene conservation were still observed after removing these receptors from the analysis (Supplemental Fig. S1). Examples of other mouse or human NK TM genes poorly conserved across vertebrates include Fc receptors, granulysin (*GNLY*), *CD160*, *CD244*, and *IFITM3*. In addition, among conserved protein coding genes, NK-cell–specific genes consistently had lower sequence identity across all vertebrates for TM genes but not for cytoplasmic ones (Supplemental Fig. S2).

The ratio between nonsynonymous and synonymous substitutions (*d*_N_/*d*_S_ ratio) of one-to-one orthologs between human and mouse can provide a good estimation of the evolutionary pressure acting on a gene. Our results indicate that NKs’ TM genes evolve faster (i.e., present higher *d*_N_/*d*_S_ values) compared with T cells’ TM genes (Supplemental Fig. S3).

As expected, the lowest conservation for all immune-cell-type–specific genes was observed in the lamprey (Fig. 1) since these organisms possess a distinct adaptive immune system (Guo et al. 2009).

To further explore the conservation of immune cell types’ specific genes and expand our understanding of immune cell populations in an evolutionary distant nonmammalian species, we set out to profile immune cell populations in zebrafish.

### Single-cell transcriptomics of zebrafish *lck*^+^ lymphocytes reveal three distinct cell populations corresponding to T cells, NK-like cells, and myeloid-like cells

As reliable antibodies to isolate pure immune cell populations in fish species are not available, we used single-cell transcriptome analysis of zebrafish *Tg(lck:GFP)* cells. This transgenic line expresses GFP under the control of the lymphocyte-specific protein tyrosine kinase (*lck*) promoter, and it was proposed to be mainly restricted to zebrafish T cells (Langenau et al. 2004). However, as *Lck* in mouse and humans is expressed in both T and NK cells, we speculated that its expression pattern could be conserved in ray-finned fish. *Tg(lck:GFP)* zebrafish may therefore provide an ideal model to investigate the large difference in conservation between T- and NK-cell–specific genes observed in mammalian species. To simultaneously obtain information about cell morphology and high-quality gene expression profiles, we used high-throughput single-cell RNA sequencing combined with FACS (fluorescent-activated cell sorting) index sorting analysis of two adult zebrafish (3- and 10-mo-old) spleen-derived *lck:GFP* cells.

**Figure 2.**
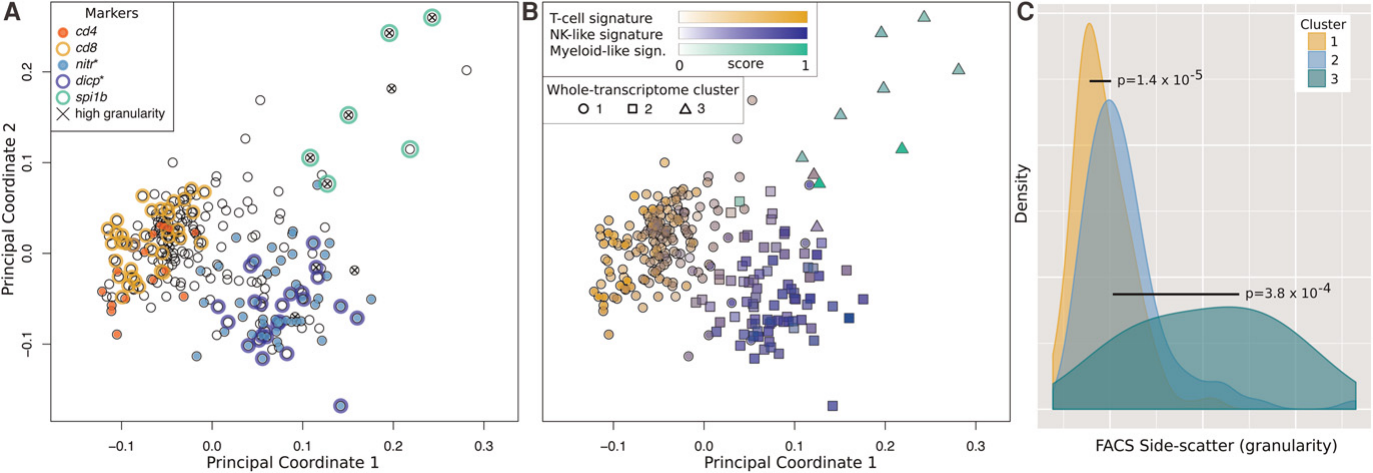
(*A*) Multidimensional scaling of zebrafish *lck*^+^ single-cell transcriptomes. Expression of known immune marker genes is depicted (using an expression threshold of five transcripts per million [TPM]). High granularity depicts cells with high side scattered light (top 5%). (*B*) Expression of T, NK-like, and myeloid cells’ transcriptional signatures (colors) together with the results of whole-transcriptome unsupervised clustering (shapes). (*C*) Distribution of side scattered light (proxy for cellular granularity) for cells in each cluster.

We first generated and sequenced libraries from 278 single GFP^+^ cells isolated from the spleen of two different fish from a different clutch and different age (see Methods). Following quality controls (see Methods) (Supplemental Fig. S4), 15 cells were removed, and gene expression profiles for the remaining 263 cells were generated. Average single-cell profiles showed good correlation with independent bulk samples (Pearson's correlation coefficient [PCC] = 0.82) (Supplemental Fig. S5). Correlations between single-cell gene expression profiles were used to calculate cell-to-cell dissimilarities (see Methods), and these were represented into low-dimensional space using classical multidimensional scaling (MDS; see Methods). Interestingly, a clear cell subpopulation structure emerged (Fig. 2A) showing three distinct cell groups with distinct expression of known immune markers such as *cd8* and *cd4* for T cells, *nitr* and *dicp* for teleost fish NK-like cells (Yoder et al. 2004), and *spi1b* for myeloid cells (see Methods) (Ward et al. 2003). Next, we performed differential expression analysis of the cells expressing these markers to derive broader zebrafish T-cell, NK-like, and myeloid-like cell signatures (see Methods) (see Supplemental Table S1) and determine whether absence of expression of specific markers in many cells was mainly due to technical limitations of single-cell RNA-seq technology (i.e., high transcript dropout rates). Indeed, the vast majority of the cells displayed clear expression of one of the gene signatures (Fig. 2B), suggesting that they belong to one of the three postulated populations (T, NK-like, and myeloid-like cells). Importantly, the three cell populations visible in Figure 2A are consistent with unsupervised whole-transcriptome clustering (see Methods) (Fig. 2B; Supplemental Fig. S6E,F). We named the obtained Clusters 1 (T-cells), 2 (NK-like cells), and 3 (myeloid-like cells).

**Figure 3.**
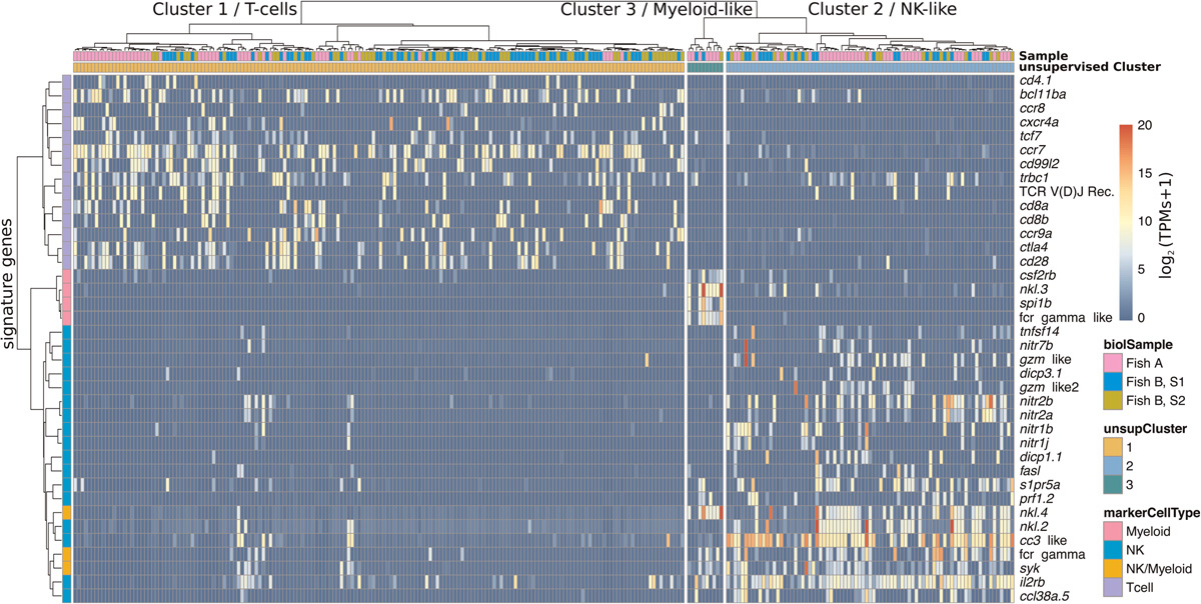
Heatmap showing the expression levels of important differentially expressed marker genes. Columns and rows represent cells and marker genes, respectively. Colors of the columns show the plates (*top* row) and the assigned clusters for each cell based on unsupervised whole-transcriptome clustering (second row; dendrogram shown on *top*). Colors of the rows (*left*-most column) indicate the known function of marker genes based on the literature (T-cell, NK, or myeloid marker). The heatmap color scale indicates the log_2_ TPMs (see Methods). Apart from a few cells in the T-cell cluster that show expression of NK markers, the unsupervised whole-transcriptome clustering is very well recapitulated by expression of known and putative cell-type markers.

To further support the hypothesis that most cells in Cluster 1 are bona fide T cells, we adapted a recent method for detection of V(D)J recombination events of the *TCR* locus (see Methods) (Stubbington et al. 2016). With a median of only 0.64 million gene-mapped reads per cell, we were able to unambiguously detect V(D)J recombination events in 27 cells (Fig. 3; Supplemental Fig. S10). Occurrence of V(D)J recombination was associated with Cluster 1 (*P* < 0.01; see Methods), which provides additional genomic evidence of the T-cell identity. As expected, V(D)J recombined segments were also strongly associated with expression of the *T cell receptor beta constant 1* (*trbc1*; *P* < 10^−5^) (Fig. 3). Interestingly, *cd8* and *cd4* displayed mutually exclusive expression (as expected for mature T cells) (Figs. 2A, 3) and *cd4*^+^ and *cd8*^+^ cells clearly separated when low-dimensional projection was restricted to cells from Cluster 1 (Supplemental Fig. S8).

In addition to *nitr* and *dicp* genes, the second cluster expressed NK lysins with high specificity (in particular *nk-lysin tandem duplicate 2* and *4*) (see Fig. 3), which have been recently proposed to mark a distinct population of NK-like cells and are up-regulated in *recombination activating gene 1*–deficient (*rag1*^−/−^) zebrafish (Pereiro et al. 2015; Moore et al. 2016). This further supported our hypothesis that cells in this cluster mainly correspond to a zebrafish equivalent of mammalian NK cells.

The clustering structure of our fish immune cells was further validated in a set of more than 300 single cells from a third fish and additional cells from the first fish, where despite much lower coverage due to external RNA contamination of the samples, the separation between cells expressing the different markers (*cd4*, *cd8*, *nitr*, *dicp*, and *spi1b*) is clearly visible (Supplemental Fig. S7; Supplemental Methods).

In addition to distinct transcriptional states, FACS analysis revealed that cells in different clusters differ in their light scattering properties (Fig. 2C). In particular, side scattered light (SSC), which is positively correlated with subcellular granularity or internal complexity, was 25% higher in Cluster 2 than in Cluster 1 (Wilcoxon rank-sum test *P* = 1.4 × 10^−5^). This is consistent with NK-like cells possessing dense cytoplasmic granules (Yoder and Litman 2011). In addition, SSC of cells in Cluster 3 was 203% higher than in the other two clusters together (*P* = 1.6 × 10^−5^). The high granularity of cells in Cluster 3 further supports the hypothesis that these cells originate from a subpopulation of *lck*^+^ myeloid cells, such as granulocytes (for similar findings in mammals, see Gibbings and Befus 2009).

Since *lck:GFP*^+^ cells were sorted randomly from spleen, the number of cells within each cluster could be used as an estimate of the frequency of each cell type in the spleen in zebrafish. Similar to what is known from the mouse (including *Lck:gfp* transgenic mice) (Shimizu et al. 2001) and humans, T cells were more frequently found (65.4% of cells fall in Cluster 1) than NK-like cells (30.8% of cells fall in Cluster 2).

### Differential expression analysis identifies both known and novel genes specific for each cell type

To identify additional genes specific for each cell population, we performed differential expression analysis of each cluster versus the other two (see Methods) (Supplemental Table S2).

The T-cell signature genes *cd4*, *cd8a*, *ctla4*, and *cd28*; the transcription factors *bcl11b* and *tcf7*; and the cytokine/chemokine receptors *il10rb*, *ccr7*, *ccr9*, and *cxcr4* were within the most differentially expressed genes in Cluster 1 (Fig. 3). We also identified many T-cell–specific genes that were uncharacterized or did not have an informative name or description in the zebrafish genome for which we assigned a putative name, based on sequence similarity searches. These included the *cd8* beta chain (ENSDARG00000058682) whose expression is highly correlated with the alpha chain *cd8a* within Cluster 1 (Fig. 3), *cd28* (ENSDARG00000069978), and an uncharacterized Ig-like protein (ENSDARG00000098787) related to CD7 antigen (Fig. 3).

Mammalian NK cells kill target cells by either of two alternative pathways: the perforin/granzyme secretory pathway or the death receptor pathway. Our analysis revealed differential expression of several members of both pathways in Cluster 2. For instance, differential expression of known innate immune receptors *nitr* and *dicp*, *syk* kinase, multiple granzymes, perforins, and NK lysins is linked with activation of the secretory pathway, whereas differential expression of *Fas ligand* (*faslg*) indicates activation of the death-receptor-ligand pathway (Dybkaer et al. 2007) in NK-like cells (Fig. 3; Supplemental Table S2). Expression of these genes further shows that zebrafish presumably resting NK-like cells transcriptionally resemble effector CD8 T cells, as observed in mammals (Bezman et al. 2012).

We also observed a high expression level of multiple cytokines and cytokine receptors. For example, differentially expressed genes in Cluster 2 included the *sphingosine-1-phosphate receptor 5a* (*s1pr5a*, whose homolog in mammalian NK cells is required for homing), the *interleukin 2 receptor beta* (IL2 induces rapid activation of mammalian NK cells), *tnfsf14* (*tumor necrosis factor [ligand] superfamily, member 14*), and chemokines of the families *ccr38* and *ccr34*. In addition, we detected differential expression of putative activating NK receptors’ adaptors (ITAMs), *Fc receptor gamma subunit* (*fcer1g*), *hematopoietic cell signal transducer* (*hcst*), *CD247 antigen like* (*cd247l*), and multiple putative transcription factors (Supplemental Table S2). Finally, within the top differentially expressed genes of these NK-like cells, we found putative homologs of mammalian granzyme B that is expanded in ray-finned fish genomes (ENSDARG00000078451, ENSDARG000 00093990, ENSDARG00000055986), and many uncharacterized putative immunoglobulin-like receptors and cytokines, such as immunoglobulin V-set domain-containing proteins or interleukin-8-like domain-containing chemokines (Table 1). Altogether, these results add confidence in our proposed classification of these cells as putative fish NK-like cells.

**Table 1.**
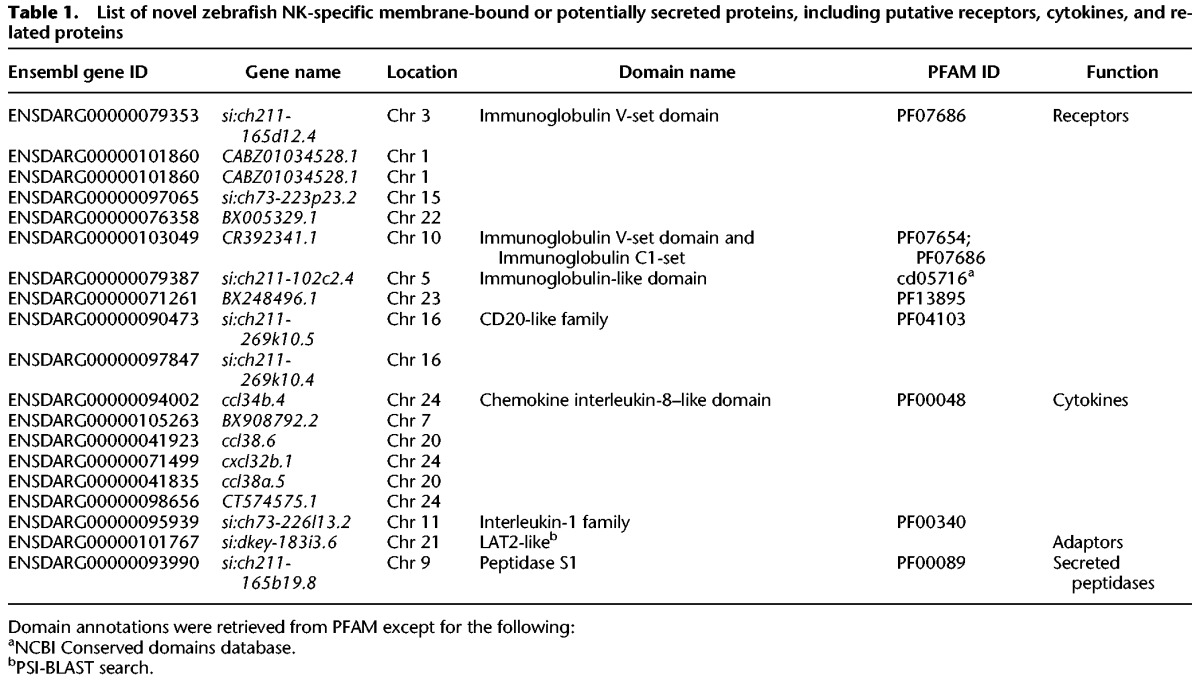
List of novel zebrafish NK-specific membrane-bound or potentially secreted proteins, including putative receptors, cytokines, and related proteins

Regarding cells in Cluster 3, the small number of cells within this cluster limited the power of differential expression analysis. Nevertheless, within the most differentially expressed genes in Cluster 3, we found two myeloid lineage–specific genes: the transcription factor *spi1b* and the granulocyte/macrophage *colony-stimulating factor receptor beta* (*csf2rb*). Other differentially expressed genes included *Fc receptor gamma subunit–like* (*fcer1gl*), *hck*, a member of the Src family of tyrosine kinases mostly expressed by phagocytes in mammals and potentially implicated in signal transduction of Fc receptors and degranulation (Guiet et al. 2008), complement factor B (*zgc:158446*), and *id2* (a transcription factor interacting with *spi1b*) (see Fig. 3).

We next compared differentially expressed genes in each cell population to human transcriptomic data of homogeneous FACS sorted immune cells (Chambers et al. 2007; Watkins et al. 2009). For genes differentially expressed in Cluster 1, our results show a significant enrichment in genes differentially expressed in human T cells (*P* = 0.008; see Methods). Similarly, the comparison of differentially expressed genes in Cluster 2 with human gene expression data confirmed a significant enrichment in NK-specific genes (*P* = 0.009), thus supporting the conservation of a core transcriptional program between mammalian and zebrafish NK-like cells (see Methods). Finally, differentially expressed genes in Cluster 3 were weakly enriched in human myeloid-specific genes (odds ratio = 5.2, *P* = 0.06; see Methods).

### Functional divergence of duplicated immune genes in zebrafish

**Figure 4.**
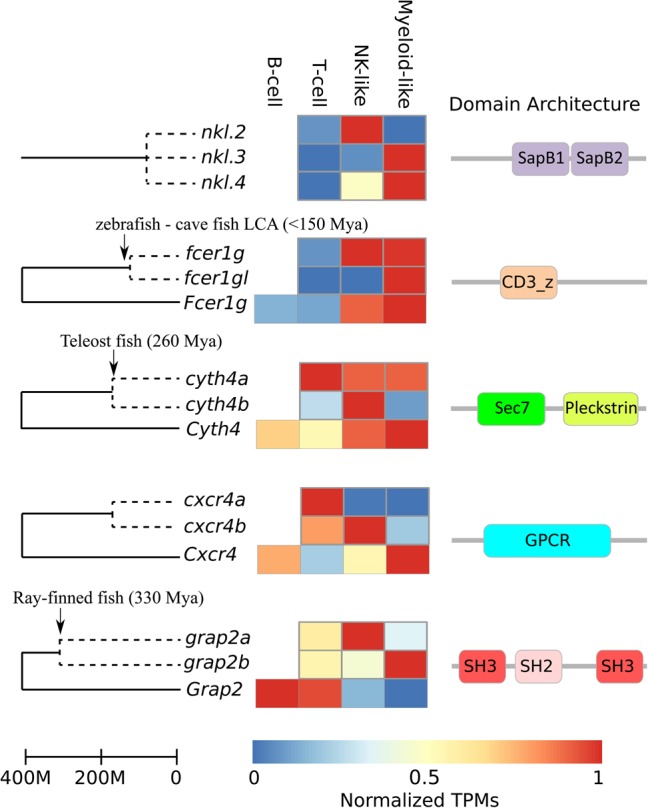
Examples of ray-finned fish–specific duplicated genes with diverged expression patterns. For genes with known mammalian orthologs, the expression in mouse is shown *below*. Estimated times of gene duplication are indicated with arrows. Domain architectures were retrieved from PFAM. (CD3_z) T-cell surface glycoprotein CD3 zeta chain; (GPCR) G-protein coupled receptor; (SH2/3) Src-homology 2/3.

Gene duplication is a common event in eukaryotic genomes and plays a major role in functional divergence. To systematically explore this functional divergence in fish immune genes, we collected all duplicated genes pre- and post-ray-finned fish speciation (see Methods). Interestingly, genes more recently duplicated (ray-finned fish specific) show lower expression in our data set. For example, 53% of prespeciation duplicated genes showed expression in *lck*^+^ cells, compared with 41% of post-speciation duplicated paralogs. As expected, prespeciation duplicated immune genes were more likely (94%) to functionally diverge (i.e., show differential expression in the immune populations; see Methods) compared with the more recent post-speciation paralogs (62%). Ray-finned fish–specific duplicated genes with conserved expression patterns included, for instance, the NK receptors *nitr*, which although expanded in zebrafish, have kept their cell-type specificity*.* In contrast, other fish-specific paralogs show distinct expression, suggesting possible neofunctionalization events (see Fig. 4). Notably, NK-lysins (*nkl.2*, *nkl.3*, *nkl.4*) provide an interesting example of such recent functional divergence. In our data, *nkl.4* was expressed in both myeloid- and NK-like cells. However, *nkl.3* was only expressed in myeloid-like cells, while *nkl.2* expression was restricted to NK-like cells (Figs. 3, 4). A second example of neofunctionalization is the *Fc receptor gamma subunit* (*fcer1g*), which in mouse (*Fcer1g*) and human (*FCER1G*), is highly expressed in myeloid and NK cells (Kikuchi-Maki et al. 2005). In zebrafish *lck*^+^ cells, Fc receptor gamma subunit (*fcer1g*) was expressed in myeloid- and NK-like cells, while its paralog *Fc receptor gamma subunit–like* (*fcer1gl*) expression was restricted to the myeloid-like cells (Fig. 4). Other examples of such neo- or subfunctionalization of recently duplicated paralogs are shown in Figure 4 and Supplemental Table S3.

### NK-specific genes show lower conservation than T-cell genes from mammals to teleost fish

**Figure 5.**
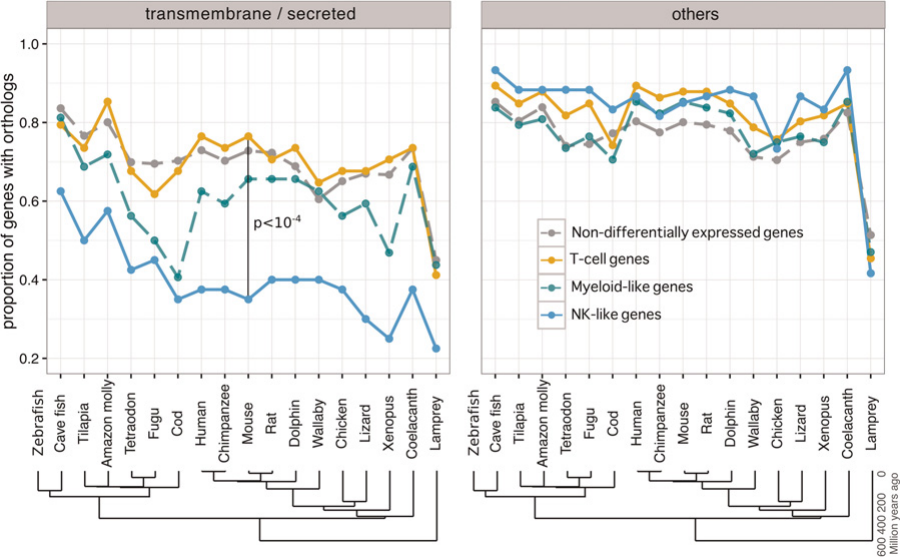
Conservation analysis of zebrafish immune genes across vertebrates. The proportion of orthologs of protein-coding genes for nondifferentially expressed genes (gray), differentially expressed genes in T cells (orange), NK-like cells (blue), and myeloid-like cells (green) are shown for both TM or secreted proteins (*left*) and other proteins (*right*).

The immune system is constantly adapting to new pathogens and changes in virulence mechanisms and, hence, is one of the most rapidly evolving biological systems in vertebrates (Kosiol et al. 2008; Fumagalli et al. 2011). To explore the evolution of the newly identified zebrafish genes specific for T, NK-like, and myeloid-like cells, we performed the same conservation analysis as in Figure 1 (see Methods). Consistently, among TM or secreted proteins, 76% of differentially expressed genes in zebrafish T cells had orthologs in mouse or human compared with only ∼36% of differentially expressed genes in zebrafish NK-like cells (*P* < 10^−4^) (Fig. 5), suggesting higher rates of gene turnover in NKs across vertebrate evolution. Among non-TM or secreted protein coding genes, the proportion of orthologs was similar between T, NK-like, and myeloid-like cell-specific genes (Fig. 5), as observed in mammalian species.

Examples of TM genes with no detected orthologs beyond teleost fish include putative chemokines (e.g., ENSDARG000000 98656), Ig-like molecules (e.g., ENSDARG00000079387), NK lysins, and the NK receptors *nitr* and *dicp* among NK-like specific genes (see also Table 1), as well as Ig-like protein coding genes (e.g., ENSDARG00000098787) among T-cell–specific genes. Although *lck*^+^ myeloid cells represent only a subpopulation of fish myeloid cells, their genes consistently show an intermediate level of conservation between T- and NK-cell–specific genes, as observed for myeloid cells in mammals (Figs. 1, 5).

When compared at the sequence identity level, the conserved TM genes specifically expressed in either T or NK/NK-like cells from human, mouse, or zebrafish had lower sequence identity than other genes across vertebrates (Supplemental Fig. S2). Moreover, as in human and mouse, zebrafish TM genes conserved in vertebrates (for which orthologs were detected) were more divergent in NK/NK-like cells than in T cells (*P* = 0.03) (Supplemental Fig. S2C). In contrast, cytoplasmic and nuclear T-cell–specific genes displayed similar sequence identity compared with other genes (Supplemental Fig. S2C).

## Discussion

The availability of fully sequenced genomes in several vertebrate species has enabled analysis of the evolution of the immune system based on orthology of known mammalian immune cell markers. However, a comparison of immune cell types at the cellular and molecular level has progressed slowly, mainly owing to the lack of suitable antibodies that mark distinct immune cell populations in the lower vertebrates. Here we used scRNA-seq of *lck:GFP* cells to characterize immune cell populations in zebrafish and examine their conservation in other vertebrate species. Our work establishes scRNA-seq as a powerful technique to study immune cell types across vertebrate species.

Analyzing single cells from two fish, we found three consistent clusters of cells, each comprising cells from both fish. The most abundant population of cells in our data set had a clear molecular T-cell signature. The cells in this cluster showed differential expression of hallmark genes important in regulation of T-cell development and signaling, suggesting a conserved transcriptional program from mammals to fish. Within this population, we were able to detect *TCR* V(D)J recombination in 22 cells (Fig. 3; Supplemental Fig. S10). Interestingly, a single *TCR* recombinant was found in each cell (Supplemental Table S6), which is consistent with allelic exclusion. Although V(D)J recombination was clearly correlated with T-cell identity, five cells with evidence of V(D)J recombination fall in Cluster 2 and three of them show clear expression NK genes. It is tempting to speculate that these cells could be NKT cells. However, in mammals, the process of TCR rearrangement first initiates in uncommitted hematopoietic progenitors before NK/DC/B/T divergence. Therefore, incomplete rearrangements are also observed in subpopulations of non-T cells, such as NKs (Pilbeam et al. 2008). This could explain the presence of V(D)J rearrangements in NK-like cells at the transcriptional level, as well as expression of single V or J segments in cells in Cluster 2 and Cluster 3. Moreover, TCRs expressed by NK T cells present a limited diversity, while here we found no evidence for preferential use of specific segments among these cells.

In mammals, *LCK* is expressed in both T and NK cells, and in our data set, one population of *lck*^+^ cells resembled NK cells. Although NK-like cells were first identified in catfish (Shen et al. 2004) over a decade ago, very little is known about the NK cell transcriptome beyond mammals. Our data revealed that the proposed bony fish NK receptors of the *nitr* family showed restricted expression in a distinct cell population of NK-like cells that also expressed granzymes, perforins, NK lysins, *Fas ligand, tnfsf14, interleukin 2 receptor beta*, the homolog of chemokine receptor CCR2, the *sphingosine-1-phosphate receptor 5a* (required for homing of mammalian NK cells), specific transcription factors, and multiple novel putative NK-specific receptors and chemokines (Table 1; Fig. 3).

Throughout evolution, animals and plants have developed complex immune defense mechanisms to combat microbial infections. However, pathogens experience strong selective pressure to evade host recognition and thus impose selective pressure on the host to re-establish immunity. As a consequence, immune-related genes have been preferential targets of positive selection in vertebrates (Kosiol et al. 2008; Yoder and Litman 2011). By using a genome-wide unbiased approach based on transcriptomic data from two mammalian and one bony fish species, we showed that a lower fraction of orthologs and lower protein sequence identity are observed for NK TM genes compared with other immune-cell-type–specific TM genes, and especially T-cell TM genes, even though T and NK cells are functionally more related (e.g., TCD8 and NK cytotoxicity upon MHCI recognition). Importantly, the trend is not only due to known NK receptors (i.e., killer cell lectin-like receptor in mouse, *KIR* in humans, and *nitr/dicp* in zebrafish) (Supplemental Fig. S1). This suggests that rapid evolution of NK TM genes is key for their function in all vertebrates. As NK genes cannot undergo somatic rearrangement, we propose that this fast evolution reflects, at least partly, a need for NK cells to possess a diverse repertoire of species-specific germline encoded receptors and associated proteins to perform their functions. In particular, both T and NK cells recognize the fast-evolving and highly polymorphic MHC molecules. While T cells do so by rearranging their *TCR* sequence, NK cells possess an expanded family of receptors. The fast evolution of these receptors may be the result of a need to adapt to MHC rapid evolutionary changes. Our observations also suggest a model of high gene turnover and faster evolution of immune TM/secreted genes but, at the same time, conservation of core cytoplasmic immune genes from zebrafish to mammalian species (Figs. 1, 5). As such, it supports zebrafish as an appropriate model organism for immune cell intracellular signaling studies.

Overall, our work expands the analysis of immune cell types and their evolution to lower vertebrates. To our knowledge, this is the first study to characterize T and NK cells at the whole-transcriptome level in a nonmammalian species and one of the first studies to analyze NK cells’ gene expression at the single-cell level (for scRNA-seq of human NK cells, see Björklund et al. 2016; for qPCR analysis of *Tg(lck:GFP)* zebrafish single cells, see Moore et al. 2016). We confirmed cell-type–specific expression of expected zebrafish T- and NK-cell genes and predicted new markers of these two cell types. We further identified cases of neofunctionalization in recent fish immune-related paralogs. Global conservation analysis revealed more rapid turnover of NK-specific TM genes compared with other immune-cell–specific, and especially T-cell–specific, genes in mammals and fish, suggesting that this is a general property of NK cells.

## Methods

### Conservation analysis of mouse and human immune cell differentially expressed genes

Orthologs of mouse and human protein-coding genes and their sequence identities, as well as TM domains and signal-peptide predictions were downloaded from BioMart/Ensembl Genes 82 (Yates et al. 2016). For genes having multiple orthologs, we considered their average sequence identity. Mouse and human NK-cell, T-cell, B-cell, granulocyte, and monocyte microarray gene expression data sets were obtained from Chambers et al. (2007) and Watkins et al. (2009). First, we prefiltered genes with low expression levels among these cell types using a threshold on normalized expression level of five for the mouse data (16,060 genes) and eight for the human data (8242 genes). CD8 and CD4 T-cell samples were merged into a T-cell group, and monocyte and granulocyte samples were merged into a myeloid cells group. We then obtained differentially expressed genes in each group compared with the others using *limma* (version 3.28.14) (Ritchie et al. 2015). Significantly differentially expressed genes (Benjamini-Hochberg adjusted *P*-value <0.01) were ordered based on expression fold-change, and the top 100 genes unique for each cell type were selected as “signature genes” for downstream analysis (Supplemental Tables S4, S5). Results were robust to different cut-offs for the top N differentially expressed genes (Supplemental Methods; Supplemental Fig. S9). Human and mouse *d*_N_/*d*_S_ ratios (Supplemental Fig. S3) of one-to-one orthologs between these two species were obtained from Ensembl version 82. The two protein groups enriched in (1) TM and secreted proteins and (2) cytoplasmic and nuclear proteins were defined based on the presence of predicted TM domains and/or signal peptide. Statistical significances of differences in sequence identity and *d*_N_/*d*_S_ differences were assessed using Wilcoxon rank-sum tests. Statistical significances of differences in proportion of orthologs were assessed as follows: (1) in a specific species (e.g., “human” point in Supplemental Fig. S2A) by comparison against a null-model distribution generated from 10,000 random permutations of gene/cell-type specificity class pairs, and (2) globally across all species (as in Supplemental Fig. S2C) using paired Wilcoxon rank-sum test (to evaluate “consistency” of the difference in conservation patterns between two cell types).

### Zebrafish strains and maintenance

Wild type (Tubingen long fin) and transgenic zebrafish *Tg(lck:EGFP)* lines were maintained as previously described (Bielczyk-Maczyńska et al. 2014), in accordance with European Union regulations on laboratory animals.

### Single-cell sorting and whole-transcriptome amplification

The spleens from two heterozygote *Tg(lck:EGFP)* adult fish from a different clutch and at different ages (3 and 10 mo) and one adult wild-type fish were dissected and carefully passed through a 40-μm cell strainer using the plunger of a 1-mL syringe, and cells were collected in cold 1× PBS/5% FBS. The nontransgenic line was used to set up the gating and exclude autofluorescent cells. Propidium iodide (PI) staining was used to exclude dead cells. Individual cells were sorted, using a Becton Dickinson influx sorter with 488- and 561-nm lasers (Schulte et al. 2015) and collected in single wells of 96-well plates containing 2.3 µL of 0.2% Triton X-100 supplemented with 1 U/µL SUPERase·In RNAse inhibitor (Ambion). The size, granularity, and level of fluorescence for each cell were simultaneously recorded. Seven wells were filled with 50 cells each from the second fish to compare single-cell with bulk RNA-seq (Supplemental Fig. S5). The Smart-seq2 protocol (Picelli et al. 2014) was used to amplify the whole-transcriptome and prepare libraries. Twenty-five cycles of PCR amplification were performed. A similar analysis was performed on two additional plates of the first fish and four plates from a third fish, including five wells with 50 cells each (see Supplemental Methods; Supplemental Fig. S7).

### Single-cell RNA-seq data processing

Following Illumina HiSeq2000 sequencing (125-bp paired-end reads), single-cell RNA-seq reads were quality trimmed and cleaned from Nextera adaptor contaminant sequences using BBduck (http://sourceforge.net/projects/bbmap) with parameters minlen=25 qtrim=rl trimq=10 ktrim=r k=25 mink=11 hdist=1 tbo.

An average of 2.1 million paired-end reads were obtained per single cell (Supplemental Fig. S4B). Next, gene expression levels were quantified as E_*i,j*_ = log_2_(TPM_*i,j*_ + 1), where TPM_i,j_ refers to TPM for gene *i* in sample *j*, as calculated by RSEM 1.2.19 (Li and Dewey 2011). RSEM (which uses Bowtie 2.2.4 for alignment) was run in paired-end non-strand-specific mode with other parameters by default using the latest zebrafish genome assembly and transcript annotations (GRCz10/GCA_000002035.3) combined with eGFP sequence appended as an artificial chromosome. For each single cell, about 0.8 million reads on average (with a median of 0.65 million) were mapped to the transcriptome (Supplemental Fig. S4A). On average, 1240 expressed genes per cell were detected (Supplemental Fig. S4C). Cells having fewer than 500 detected genes or fewer than 10,000 reads mapped to transcripts were excluded from further analyses.

### Transcriptome dimensionality reduction, batch effect removal, and cell clustering

In order to visualize cell heterogeneity at the transcriptomic level, we used classical MDS (i.e., principal coordinates analysis, as implemented in R's *cmdscale* function) for dimensionality reduction (Fig. 2A; Supplemental Fig. S6A). MDS attempts to preserve distances between points generated from any dissimilarity measure. PCCs between full transcriptional profiles were used to define cell-to-cell similarities, and 1 − PCC was then used as MDS's input dissimilarity measure.

To correct for batch effects and remove unwanted variation between the first and second fish, we used the ComBat function from R Bioconductor's *sva* package (Parker et al. 2014). After this procedure, variation between individuals was minimal (Supplemental Fig. S6G).

Similar low-dimensionality projection was obtained using zero inflated factor analysis (ZIFA) (Pierson and Yau 2015; downloaded from https://github.com/epierson9/ZIFA in July 2016) that explicitly models gene drop-out events (Supplemental Fig. S6B). As input to ZIFA we used ComBat-adjusted log_2_ (TPMs + 1) having a minimum variance across cells of one (6350 genes passed this filter). As the batch effect adjustment can produce negative expression values and ZIFA requires all values to be positive, we set all negative values to zero. ZIFA was run in the fast “blocks” mode with *k* = 5.

To identify different cell populations, we first defined, based on literature, minimal sets of marker genes for candidate T cells (*cd4*, *cd8a*, *cd8b*, *cd28*, and *ctla4*), NK-like cells (all members of *nitr*, *dicp* and *nk-lysin* families), and myeloid cells (*spi1b*/*pu*.*1*). We calculated a score for each set of marker genes [mean log_2_ (TPMs + 1)] and assigned identities to cells having a score higher than one in only one of the three sets, yielding 91 T cells, 44 NK-like cells, and five myeloid cells (Fig. 2A). We next performed differential expression analysis between these three sets of cells, using SCDE R package v1.99 (Kharchenko et al. 2014). Differentially expressed genes with a log_2_ fold-change above two and an adjusted *z*-score above three were defined as extended zebrafish cell-type signatures (Supplemental Table S1). These extended signatures were used to color all cells (Fig. 2B). For simplicity, we standardized scores to range from zero to one. In addition, we performed hierarchical clustering using Ward's criteria (as implemented in R's *hclust* using *Ward.D2* method) applied on the first four principal coordinates generated by the MDS. The choice of the components was based on the eigenvalue decomposition of the MDS (Supplemental Fig. S6C). Eigenvalues decrease very smoothly after the fourth component, i.e., contributing less significantly to the overall variability. The number of clusters (Fig. 2B; Supplemental Fig. S6E,F) was determined by maximizing the mean silhouette coefficient (Supplemental Fig. S6D).

### *TCR* reconstruction

All four *TCR* loci (*alpha*, *beta*, *delta*, and *gamma*) and Rag-dependent V(D)J recombination are found in zebrafish (Langenau and Zon 2005). However, only the *beta chain* locus was fully annotated (Meeker et al. 2010). To explore TCR recombination in our immune cell populations, we adapted the recent method of Stubbington et al. (2016). Synthetic *beta chain* sequences containing all possible combinations of the 52 V and 33 J germline segments were generated, with the addition of 20 N ambiguity bases in the 5′ end, seven N's between the V and J segments, and 50 N's at the 3′ end to account for unknown leader, possible D, and constant sequences, respectively. RNA-seq reads from each cell were aligned against the collection of synthetic *TCR beta chain* sequences independently using the Bowtie 2 aligner (Langmead and Salzberg 2012), with low penalties for introducing gaps into either the read or the reference sequence or for aligning against N nucleotides (parameters ‘−no­unal −k 1 −np 0 vrdg 1,1 −rfg 1,1’). Next, reads aligning to synthetic sequences were used as input to the Trinity RNA-seq assembly software (Grabherr et al. 2011) using its default parameters for de novo assembly. Contigs assembled by Trinity were used as input to NCBI IgBlast 1.4 (Ye et al. 2013) using the parameters ‘−qcov_hsp_perc 90 −evalue 0.001 −ig_seqtype TCR −perc_identity 99’ and providing zebrafish V, D and J segments, and the resulting output was processed with a custom parsing script. Contigs with no stop codons and matching both a V and a J segment with at least 90% sequence identity against corresponding germline segments, as well as where at least 90% of the germline segment was recovered, were considered evidence for *TCR beta chain* V(D)J recombination.

### Differential expression analysis between cell clusters and marker gene discovery

Estimated gene counts obtained from RSEM were used as input for *SCDE* R package v1.99 (Kharchenko et al. 2014) that explicitly accounts for the high rate of dropout events in scRNA-seq. Differential expression between each cluster versus the other two was assessed using 500 randomizations (Supplemental Table S2).

To assess transcriptional conservation between mammalian and zebrafish immune cell types, we used the previously defined sets of human top 100 differentially expressed genes in T, NK, and myeloid cells. We then compared the proportion of zebrafish genes with orthologs in T-cell, NK-cell, and myeloid signature genes within the differentially expressed genes in each cluster versus nondifferentially expressed genes. Statistical significance was assessed using Fisher's exact test. Analyses were performed in R version 3.3.1 (R Core Team 2016). The heatmap shown in Figure 3 was produced using “Pheatmap” package (https://CRAN.R-project.org/package=pheatmap), using “correlation” distance with “Ward.D2” criteria to cluster rows, and whole-transcriptome distance (as for Fig. 2) for clustering columns.

### Expression analysis of duplicated immune genes in zebrafish

A list of paralogs in zebrafish was obtained from Ensembl Compara GeneTrees (version 82) (Vilella et al. 2009). We defined two groups of protein coding genes: (1) 14,342 genes that underwent “recent” duplication, whose most recent common ancestor was mapped to ray-finned fish (Actinopterygii) or any of its child nodes (Neopterygii, Otophysa, Clupeocephala, *Danio rerio*); and (2) 19,499 genes that underwent “early” duplication, where their most recent common ancestor was mapped to bony vertebrates (Euteleostomi) or any of its parent taxa (Bilateria, Chordata, Vertebrata). Many of these genes suffered multiple duplication events both before and after the fish common ancestor. Therefore, to compare differences in expression between these two groups, we did not include the set of overlapped genes and obtained 3235 unique recently duplicated genes and 8609 unique early duplicated genes. From these, 1315 (41%) and 4569 (53%) were detected in our data (genes with >0 TPM in at least 1% of the cells).

For the analysis of expression pattern divergence, we searched pairs of paralogs where both genes show some specific expression pattern (therefore, likely to have an immune-related function) according to one of the following criteria: (1) within the top 100 differentially expressed genes in Cluster 1, Cluster 2, or Cluster 3; (2) within the top 100 differentially expressed genes in Cluster 2 and Cluster 3 versus Cluster 1 (i.e., depleted in Cluster 1), Cluster 1 and Cluster 3 versus Cluster 2 (i.e., depleted in Cluster 2), or Cluster 1 and Cluster 2 versus Cluster 3 (i.e., depleted in Cluster 3); or (3) expressed in all the three clusters (in at least 10% of the cells of each cluster). In the latter case, we only considered pairs of paralogs where only one gene is expressed in the three clusters, and the second is either specifically expressed or depleted from the major Clusters 1 or 2. Pairs of paralogs where both genes are expressed in all three clusters were not considered since most of them are not immune-related genes, while Cluster 3 is too small to accurately assess enrichment/depletion.

Next, we identified cases where both paralogs belong to the same expression pattern group (duplicate genes with conserved expression pattern) and cases where they differ (cases of neofunctionalization due to different expression patterns). For recently duplicated genes, we found 23 pairs with distinct expression patterns and 14 pairs with the same expression patterns (i.e., 62% of paralogs’ neofunctionalization), while for early duplicated genes, we found 121 pairs with distinct patterns and eight pairs with the same expression patterns (i.e., 94% of paralogs’ neofunctionalization), as shown in Supplemental Table S3.

### Gene sequence conservation analysis of zebrafish differentially expressed genes

Orthologous genes of zebrafish in vertebrate species and their sequence identities were downloaded from BioMart/Ensembl Genes 82. For comparisons between differentially expressed genes between Cluster 1 (T cells), Cluster 2 (NK-like cells), and Cluster 3 (myeloid-like cells), we chose the top 100 differentially expressed genes after filtering by *Z*-score >1 and sorting by fold-change (Supplemental Table S2), although results were robust to different cut-offs (Supplemental Methods; Supplemental Fig. S9). To assess orthologs’ conservation among nondifferentially expressed genes, we first excluded lowly expressed genes from the analysis (those where its expression level E was below the global mean of 0.46). The reason for this is that we observed a bias of higher gene conservation among highly expressed genes compared with lowly expressed genes. After this filter, conservation of differentially expressed genes could be compared with that of nondifferentially expressed (but having equivalent expression levels across all cells) genes as in Figure 5. Supplemental Table S4 shows, for all analyzed genes, the gene sequence identity (or their average, in case of multiple orthologs) shared with orthologs in the vertebrate species analyzed.

## Data access

Raw and processed sequence data sets from this study have been submitted to ArrayExpress (https://www.ebi.ac.uk/arrayexpress/) under accession number E-MTAB-4617.

## Supplementary Material

Supplemental Material
