# Supplementary material for: Single-cell transcriptome analysis of fish immune cells provides insight into the evolution of vertebrate immune cell types
Source: Genome Res. 2017 Mar;27(3):451–61. doi: 10.1101/gr.207704.116 (PMC5340972; doi:10.1101/gr.207704.116)
Supplement: Supplemental Material [file supp_27_3_451__index.html]

Single-cell transcriptome analysis of fish immune cells provides insight into the evolution of vertebrate immune cell types — Single-cell transcriptome analysis of fish immune cells provides insight into the evolution of vertebrate immune cell types — Supplemental Material 

# Single-cell transcriptome analysis of fish immune cells provides insight into the evolution of vertebrate immune cell types

## Supplemental Material

- Supplemental\_Table\_S1.xlsx
- Supplemental\_Table\_S2.xlsx
- Supplemental\_Table\_S3.xlsx
- Supplemental\_Table\_S4.xlsx
- Supplemental\_Table\_S5.xlsx
- Supplemental\_Table\_S6.xlsx
- Supplemental\_Material.docx
