# Supplementary material for: Single-cell transcriptome analysis of fish immune cells provides insight into the evolution of vertebrate immune cell types
Source: Genome Res. 2017 Mar;27(3):451–61. doi: 10.1101/gr.207704.116 (PMC5340972; doi:10.1101/gr.207704.116)
Supplement: Supplemental Material [file supp_gr.207704.116_Supplemental_Material.docx]

**Supplementary Data**

**Single-cell transcriptome analysis of fish immune cells provides insight into the evolution of vertebrate immunity**

Santiago J. Carmona^1,2^, Sarah A Teichmann^3,4^, Lauren Ferreira^4,5,6^, Iain C. Macaulay^7^, Michael JT Stubbington^3^, Ana Cvejic^4,5,6^, David Gfeller^1,2^

^1^Ludwig Center for Cancer Research, University of Lausanne, Lausanne, Switzerland

^2^Swiss Institute of Bioinformatics (SIB), 1015 Lausanne, Switzerland

^3^European Molecular Biology Laboratory European Bioinformatics Institute, Wellcome Trust Genome Campus, Hinxton, Cambridge, UK

^4^Wellcome Trust Sanger Institute, Wellcome Trust Genome Campus, Cambridge, UK

^5^Department of Haematology, University of Cambridge, Cambridge, UK

^6^Wellcome Trust – Medical Research Council Cambridge Stem Cell Institute, Cambridge, CB2 1QR, UK

^7^Sanger Institute–EBI Single-Cell Genomics Centre, Wellcome Trust Genome Campus, Hinxton, Cambridge, UK

**Supplementary Methods**

1. **Additional replicates**

Two replicates (i.e., 96-well plates) containing *lck:gfp* cells from the first fish (black points in Supplemental Fig. 6G) as well as four other plates with cells from a third *lck:gfp* transgenic fish were prepared and sequenced with the same protocol. Due to contamination in this third batch of 6 plates prepared to assess reproducibility, only about 9% of the reads could be mapped on average to the zebrafish genome. Despite the lower quality of these plates, the three clusters corresponding to T-cell, NK and myeloid-like cell clusters could be reproduced (Figure S7). MDS was performed on these using the set of NK, T-cell and myeloid-like differentially expressed genes derived from the unsupervised analysis of the good plates. Marker genes *cd4*, *cd8a*, *nitr*’s, *dicp*’s and *spi1b* were excluded from the list and only used to show separation of cell types. Figure S6 shows the MDS plots for each of the additional plates. For cells from the third fish (Fig. S6 C to F), a more restrictive gating on side scatter (SSC) was applied in FACS index sorting to remove highly granular cells. This effectively excluded the cluster of myeloid-like cells. In replicates from the first fish (Fig. S6 A and B) where the 3 cell clusters were present, SSC pattern (not shown) was the same as observed for the original plate (Figure 2 B).

1. **Sensitivity of gene conservation analysis to different thresholds**

To simplify gene expression comparisons across species, cell types and datasets from different studies, we selected the top 100 most differentially expressed genes in each case. However, we verified that similar results on gene conservation are obtained when using different cut-offs. Supplemental Figure S9 shows the proportion of orthologs (upper panels) and sequence identity (lower panels) of zebrafish NK and T-cell specific genes in mouse (panels on the right) and of mouse NK and T-cell specific genes in zebrafish (panels on the left), for different numbers of most differentially expressed genes.

**Supplementary Figures**

**Figure S1** Conservation analysis of human (**A**), mouse (**B**) and zebrafish (**C**) immune cells differentially expressed genes (similar to Figure 1) after removing the NK receptor families KIR (e.g., Ly49 in mouse, KIR in human, and NITR and DICP genes in zebrafish). P-values indicate statistical significance of the difference in proportion of orthologs of NK and T-cell genes, based on 10,000 random permutations.

**Figure S2** Orthologs sequence identity of mouse (**A**), human (**B**), and zebrafish (**C**) immune cells differentially expressed genes. P-values indicate statistical significance of the difference of median sequence identity of NK and T-cell genes between human and mouse (A and B) or globally across species (C).


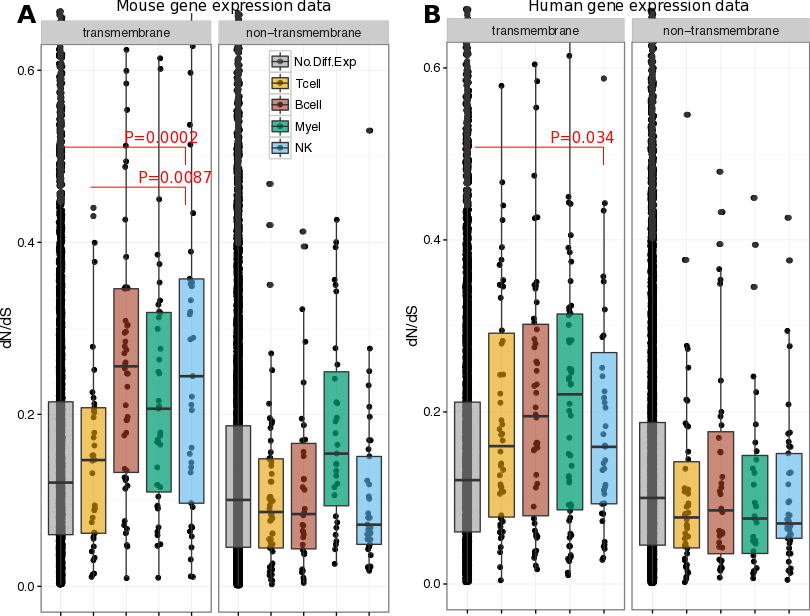


**Figure S3**: *d*_N_/*d*_S_ ratios of mouse (**A**) and human (**B**) immune cells’ specific genes.

The closer to 0 the *d*_N_/*d*_S_ ratio of a gene is, the stronger is the purifying selection operating on it. Conversely, the highest the ratio is, the faster the sequences are evolving.

**Figure S4**: Quality control of scRNA-Seq. **A**: number of reads aligned to genes. **B**: Total number of reads per cell / library. **C**: Number of detected genes (at least 1 mapped read). Horizontal bars indicate quality cut-offs used: >10,000 aligned reads (A and B) and >500 detected genes (C).

**Figure S5**: Comparison between the average gene expression across all 263 single cells and the bulk RNA-Seq sample (50 cells). Pearson’s correlation coefficient is indicated.

**Figure S6: A:** Low-dimensional representation (classical multidimensional scaling, principal coordinates 1 and 2) of cell-to-cell transcriptional dissimilarities of cells from fish 1 and 2. Similar to Figure 2A, except that expression of cell type markers is color-coded in a continuous scale (log2 TPM+1) instead of using a threshold. **B:** Low dimensional representation produced by ZIFA (principal components 2 and 3). Colors represent average expression of signature genes for T-cells, NK-like and myeloid-like cells (same as in Figure 2B). Shapes correspond to clusters obtained by unsupervised whole transcriptome hierarchical clustering (same as in Figure 2B and S6E). **C:** Eigenvalues of the MDS principal coordinates; the first four coordinates were selected to summarize cell-to-cell dissimilarities: after the fourth coordinate, their contribution decreases smoothly. **D:** Clustering mean silhouette coefficients across different number *K* of clusters. Three is the optimal number of clusters based on this criterion**. E:** MDS plot (coordinates 1 and 2) showing cluster assignment after hierarchical clustering. **F:** MDS 3D plot (coordinates 1, 2 and 3) showing cluster assignment after hierarchical clustering. **G:** MDS plot (coordinates 1 and 2) showing sample individual and batch, after batch effect removal. **H:** Boxplots showing distributions of FACS side (top) and forward scattering (bottom) for cells belonging to each of the 3 clusters.

**
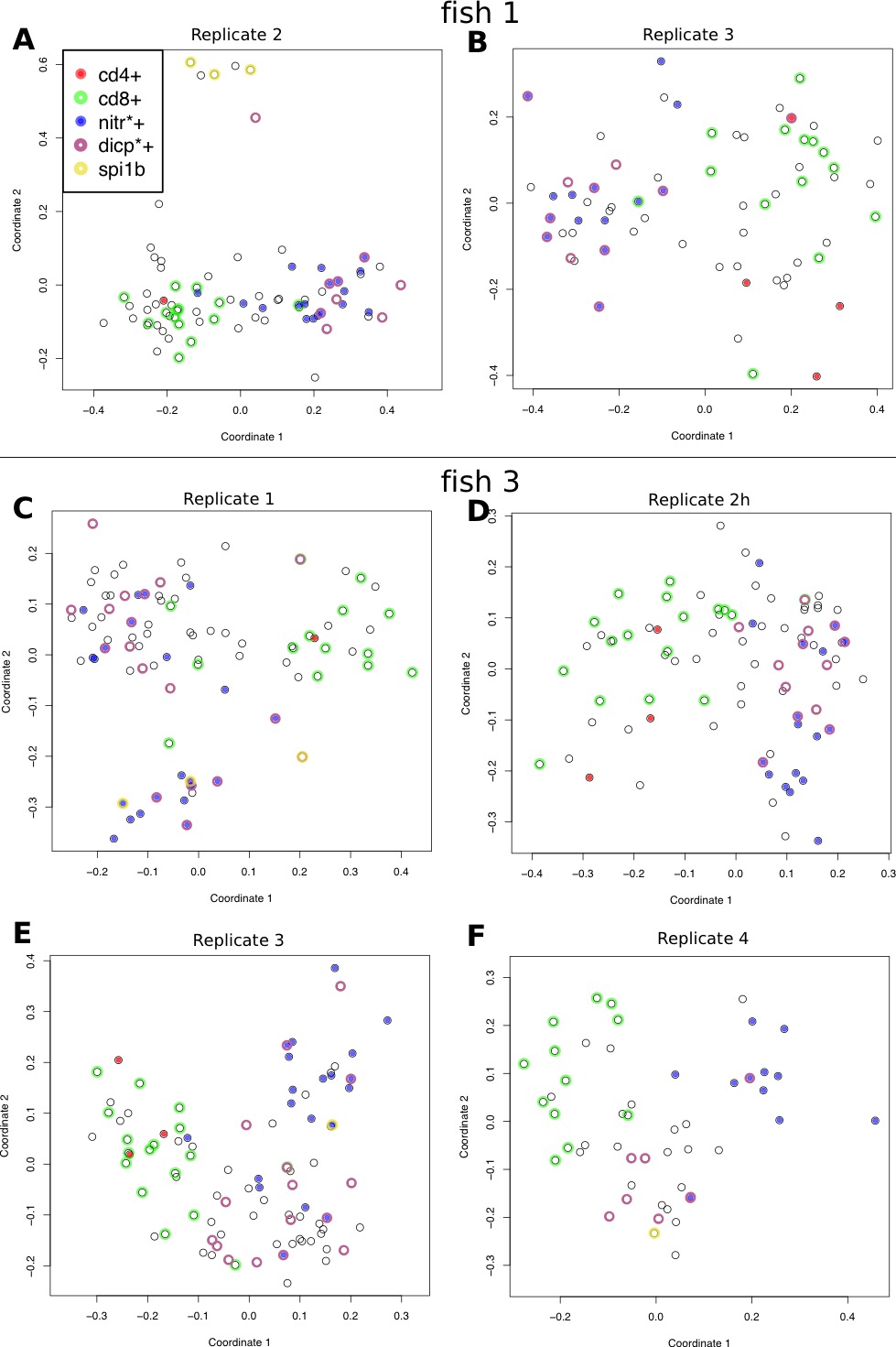
**

**Figure S7:** Low-dimensional representation (MDS) of cell-to-cell transcriptional dissimilarities for the 6 additional cell plates (**A-B**: fish 1 as in Figure 2A, **C-F**: third fish). The set of genes used in the MDS are those differentially expressed between C1 and C2 in Fig. 2A excluding those used in the plot to validate the clustering (i.e., *cd4*, *cd8a*, *nitr*’s, *dicp*’s and *spi1b*). Importantly, in the third fish, cells with high granularity were excluded in the sorting, which explains why myeloid-like cells are not often observed.

**Figure S8**: Low-dimensional representation (MDS) of cell-to-cell transcriptional dissimilarities in Cluster 1 (T-cell cluster). MDS was performed as described in Methods for all cells, except that only the 1000 most variable genes within Cluster 1 were used to calculate cell-to-cell dissimilarities. Despite fish batch effects, a clear separation is visible between *cd4*+ and *cd8*+ T cells.

**Figure S9**: Sensitivity of gene conservation analysis to different thresholds. **A:** Proportion of mouse genes (Y-axis) with orthologs in zebrafish, within the top N differentially expressed mouse NK and T-cell genes (x-axis). **B:** median percentage of identity between those orthologs **C:** Proportion of zebrafish genes (Y-axis) with orthologs in mouse, within the top N differentially expressed zebrafish NK and T-cell genes (x-axis). **D:** median percentage of identity between those orthologs.

**Figure S10:** Low-dimensional representation (classical multidimensional scaling, principal coordinates 1 and 2) of cell-to-cell transcriptional dissimilarities marking cells were events of V(D)J rearrangements were detected. Expression of the NK-like markers of the NITR family and the transcription factor Spi1 are displayed to indicate NK-like and myeloid-like cell populations, respectively. TCR recombination is strongly associated with the T-cells clusters.

**Supplementary Tables Legends**

**Supplemental Table S1**: Zebrafish T-cell, NK-like and Myeloid-like signature genes defined as differentially expressed genes between a subset of candidate T-cells, NK-like cells and Myeloid-like cells (those with clear expression of marker genes: *cd4*, *cd8a*, *cd8b*, *cd28* and *ctla4* for T-cells, *nitr*, *dicp* and *nk-lysin* families for NK-like, and *spi1b* for myeloid cells).

**Supplemental Table S2**: Differentially expression analysis of genes in zebrafish T-cell (C1 vs C2,C3; Sheet 1), NK-like (C2 vs C1,C3; Sheet 2) and myeloid-like lck+ cell clusters (C3 vs C1,C2; Sheet 3). Gene expression fold change, z-score and adjusted z-score correspond to SCDE package output. Additional columns include sequence identity against mouse and human orthologs (or ‘0’ for genes with no assigned orthologs), presence of trans-membrane domains and signal peptide. These data were obtained from Ensembl 84. Compared to the differential expression analysis performed for Table S1, this one is broader as all cells were included.

**Supplemental Table S3**: Compared expression pattern of paralogs. Pairs of paralogs were grouped by time of duplication (pre- and post- ray finned fish speciation) and expression pattern (conserved, *ie* expressed within the same cell type(s), or diverged expression patter, *ie* expressed in different cell types).

**Supplemental Table S4:** Zebrafish, Human and Mouse gene conservation across other vertebrate species. The table also indicates which genes are among the 100 most differentially expressed NK and T-cell genes in Zebrafish, Mouse and Human, and transmembrane domains and signal peptide predictions.

**Supplemental Table S5, Sheet 1**: Zebrafish top 100 differentially expressed genes in T-cells, NK-like and Myeloid cells. **Sheets 2 and 3**: Mouse and Human top 100 differentially expressed genes in T-cells, NKs, B-cells and Myeloid cells (granulocytes and monocytes).

**Supplemental Table S6**: Cells where events of V(D)J recombination of TCR beta chain were detected. Recombined V and J segments are shown.

**References**

Björklund,A.K. *et al.* (2016) The heterogeneity of human CD127(+) innate lymphoid cells revealed by single-cell RNA sequencing. *Nat. Immunol.*

Watkins,N.A. *et al.* (2009) A HaemAtlas: characterizing gene expression in differentiated human blood cells. *Blood*, **113**, e1–9.
